# Stoma associated complications after diverting loop ileostomy, end ileostomy or split stoma formation after right sided colectomy—a retrospective cohort study (StoComSplit Analysis)

**DOI:** 10.1007/s10151-024-02945-z

**Published:** 2024-06-12

**Authors:** B. Wiesler, L. Hirt, M.-O. Guenin, D. C. Steinemann, M. von Flüe, B. Müller-Stich, T. Glass, M. von Strauss und Torney

**Affiliations:** 1 Department of Visceral Surgery, Clarunis, University Digestive Health Care Center, St. Clara Hospital and University Hospital Basel, Switzerland, Basel, Switzerland; 2 University of Basel, Basel, Switzerland; 3 ChirurgieZentrum Zentralschweiz, Lucerne, Switzerland; 4 Swiss Tropical and Public Health Institute (Swiss TPH), Basel, Switzerland; 5 St. Clara Research Ltd, St. Clara Hospital, Basel, Switzerland

**Keywords:** Colorectal surgery, Right sided resection, Ostomy formation, Split stoma, End ileostomy, Loop ileostomy

## Abstract

**Background:**

For high-risk patients receiving right-sided colectomy, stoma formation is a safety strategy. Options are anastomosis with loop ileostomy, end ileostomy, or split stoma. The aim is to compare the outcome of these three options.

**Methods:**

This retrospective cohort study included all patients who underwent right sided colectomy and stoma formation between January 2008 and December 2021 at two tertial referral centers in Switzerland. The primary outcome was the stoma associated complication rate within one year.

**Results:**

A total of 116 patients were included. A total of 20 patients (17%) underwent primary anastomosis with loop ileostomy (PA group), 29 (25%) received an end ileostomy (ES group) and 67 (58%) received a split stoma (SS group).

Stoma associated complication rate was 43% (*n* = 21) in PA and in ES group and 50% (*n* = 34) in SS group (n.s.). A total of 30% (*n* = 6) of patients in PA group needed reoperations, whereas 59% (*n* = 17) in ES and 58% (*n* = 39) in SS group had reoperations (*P* = 0.07). Wound infections occurred in 15% (*n* = 3) in PA, in 10% (*n* = 3) in ES, and in 30% (*n* = 20) in SS group (*P* = 0.08). A total of 13 patients (65%) in PA, 7 (24%) in ES, and 29 (43%) in SS group achieved stoma closure (*P* = 0.02). A total of 5 patients (38%) in PA group, 2 (15%) in ES, and 22 patients (67%) in SS group had a stoma-associated rehospitalization (*P* < 0.01).

**Conclusion:**

Primary anastomosis and loop ileostomy may be an option for selected patients. Patients with end ileostomies have fewer stoma-related readmissions than those with a split stoma, but they have a lower rate of stoma closure.

**Clinical trial registration:**

Trial not registered.

**Supplementary Information:**

The online version contains supplementary material available at 10.1007/s10151-024-02945-z.

## Introduction[Update Editor corrections from '10151_2024_2945_Editor Rodrigo stoma right isde_']

Patients receiving right sided colorectal resections have a high complication rate with up to 13% when performed electively [1]. In emergency situations, this number can rise to 38% [2]. Anastomotic leakage rates for these patients range from 1% to 30% [3–6].The event of an anastomotic leakage is associated with strongly increased postoperative mortality rates [6]. Especially in emergency operations patients come with risk factors like immunosuppression, inflammatory bowel disease or malignant obstruction and are severely ill, which translates into high Sepsis related Organ Failure Assessment (SOFA) and American Society of Anesthesiology (ASA) scores [4, 7]. Several risk scores for anastomotic leakage have been developed in the past and red flags were identified [8]. When such red flags against anastomosis, like peritonitis, perforation, high use of vasopressors, or critically ill patients, are present, stoma formation is a possible safety strategy to reduce morbidity and mortality [9, 10].

The possible procedures include a primary anastomosis with upstream diverting loop ileostomy, formation of an end ileostomy with closure of the colonic stump without anastomosis or the formation of a spilt stoma.

The formation of an upstream diverting loop ileostomy allows to perform the anastomosis, which is protected by the stoma. This technique is poorly described for right sided resections [11]. As an advantage, stoma closure is generally straight forward, and leakage is rare. Nevertheless, it comes with the risk of possibly occult leak of the colonic anastomosis, which must be excluded prior to stoma closure either by endoscopy or contrast study. Another downside is the increased rate of high output stomas of up to 16%, which is higher than in the other stoma types given to the more proximal nature of the ostomy [12]. The formation of an end ileostomy and closure of the colonic thump is easily performed, and the stoma is usually less problematic in terms of stoma bag detachments or leakage due to the single opening. Nevertheless, stoma closure involves a laparotomy or laparoscopy to access the colonic stump and perform anastomosis. In case of split stoma formation, the stoma is formed by ileum and colon and the intestinal ends are drained through a common hole in the abdominal wall. Split stomas are quick to establish, and stoma closure can be performed by local detachment from the abdominal wall without extensive laparotomy. It has been shown that split stoma formation can reduce the early postoperative complication rate after colorectal surgery [13]. However, caring for a split stoma can be more complex, leading to increased need of care.

So far there is little evidence as to which strategy is the best. Consequently, the main objective of the current study is to compare the stoma associated outcome of the three safety strategies.

## Methods

### Patients

This retrospective, multicenter cohort study included all patients undergoing either elective or emergency right-sided hemicolectomy or ileocecal resection with either upstream diverting loop ileostomy with primary anastomosis, formation of an end ileostomy or split stoma between January 2008 and December 2021 in two tertiary referral centers. Patients who underwent a limited transverse colonic resection or a subtotal colectomy were excluded. The in-hospital follow-up was 1 year. For manuscript preparation the STROBE cohort reporting guidelines were used [14]. The study was approved by the local ethic committee Ethikkomission Nordwest-und Zentralschweiz (“EKNZ”) on 16 June 2020 with ethics number 2020–01496. The study was performed under the legislation of article 34 human research law of Switzerland allowing further use of routine data without individual consent.

### Data collection

Patients’ demographics, comorbidities (coronary heart disease, cerebrovascular incident, diabetes mellitus), and risk factors [oral antiplatelet therapy, oral anticoagulants, intake of immunosuppressant, previous abdominal surgery, nutritional risk score, quick sequential organ failure assessment (qSOFA) score] were collected from medical records and included in an anonymized database [15, 16]. The preoperative condition of the patients was determined using the ASA Score [17]. Wound status was defined using Centers of Disease Control (CDC) Classification [18].

According to the known intraoperative risk factors surgical data contained duration of surgery, intraoperative blood loss, experience of surgeon (consultant general or visceral surgeon, trainee visceral surgeon) and intraoperative use of vasopressors [19–21].

The patients were divided into three groups depending on stoma type. The first group contained all patients receiving an anastomosis with upstream diverting loop ileostomy (PA group). The second group was composed of patients receiving an end ileostomy (ES group). In the third group patients received a split stoma (SS group). All different types of anastomoses (hand-sewn, stapler, side-to-side, side-to-end) were included.

### Outcomes

The primary objective of the study was to assess the occurrence of any stoma-associated complication after stoma formation within one year. Stoma-associated complications were attributed to either local issues such as parastomal dermatitis or parastomal abscess, or linked to the stoma delivery rate, as seen in conditions like high output stoma and its associated consequences. The complication rate was determined using the Clavien−Dindo (C−D) Classification [22]. Secondary objective was to assess the stoma associated complication rate C−D > 2, and the overall complication rate C−D > 2. Further secondary endpoints were the in-hospital mortality and the 30-day mortality rate. Furthermore, the postoperative wound infections rate, stoma closure rate within 1 year, in-hospital complication rate according to C−D after stoma closure, stoma associated rehospitalization rate within 1 year, and reoperation rate within 1 year was assessed. From the patients’ point of view a loss of independence is considered another highly relevant aspect of care. Each patient’s habitat was assessed before and after surgery, and the rate of new care home admissions was assessed.

### Statistical analysis

Data were summarized according to stoma type with frequencies and percentages for categorical data and medians and interquartile ranges (IQR) for continuous data. The primary outcome was analyzed using univariate logistic regression. In addition, due to the small sample size and high number of potential confounders and the lack of randomization of the stoma type, a weighted logistic regression model was utilized with weights constructed as the probability of having received a split stoma. Results were presented with odds ratios (OR) and 95% confidence intervals (CI). Secondary outcomes were compared using a chi-squared test or Fisher’s exact test (when cell counts < 5). Data analysis was done using Stata version 16.1 (College Station, Texas).

## Results

### Patients

A total of 116 patients had a stoma formation after right sided colon resections during the study period. A total of 20 patients (17%) underwent primary anastomosis with diverting loop ileostomy (PA group), 29 (25%) had an end ileostomy (ES group), and 67 patients (58%) received a split stoma (SS group). Demographic data are shown in Table 1. The patients in the PA group had a lower body mass index and higher nutritional risk scores than those in the ES and SS group (Table 1). Reasons for ostomy creation in the study cohort included polymorbidity, severe reduction in overall condition, contaminated situs, revision of anastomosis, and high intraoperative use of vasopressors. The formation of a diverting loop ileostomy as part of a right colectomy is a rarely performed procedure. Supplementary Table 1 presents an overview of the reasons that led to the formation of a loop ileostomy in the study cohort.

**Table 1:** Comparison of demographic data in PA, ES, and SS group

	Total (*N* = 116)	PA group (*n* = 20)	ES group (*n* = 29)	SS group (*n* = 67)	*P* value*
Age, years					
Median (IQR)	69 (61–76)	70 (66–77)	70 (59–76)	69 (62–76)	0.44
Gender					0.32
Female	53 (46%)	11 (55%)	10 (34%)	32 (48%)	
Male	63 (54%)	9 (45%)	19 (66%)	35 (52%)	
Preoperative variables					
ASA score					
2	9 (8%)	4 (20%)	2 (7%)	3 (5%)	0.07
3	54 (47%)	11 (55%)	12 (41%)	31 (46%)	0.64
4	48 (41%)	4 (20%)	14 (48%)	30 (45%)	0.09
5	4 (3%)	0 (0%)	1 (3%)	3 (5%)	1
SOFA score					
0	81 (70%)	17 (85%)	21 (72%)	43 (64%)	0.19
1	13 (11%)	2 (10%)	2 (7%)	9 (13%)	0.78
2	15 (13%)	1 (5%)	3 (10%)	11 (16%)	0.40
3	7 (6%)	0 (0%)	3 (10%)	4 (6%)	0.35
Wound status					
I	23 (20%)	3 (15%)	4 (14%)	16 (24%)	0.53
II	28 (24%)	8 (40%)	5 (17%)	15 (22%)	0.18
III	26 (22%)	2 (10%)	7 (24%)	17 (25%)	0.38
IV	27 (32%)	5 (25%)	13 (45%)	19 (28%)	0.22
Previous abdominal surgery					
No	47 (41%)	8 (40%)	9 (31%)	30 (45%)	0.45
Yes, laproscopic	21 (18%)	3 (15%)	6 (21%)	12 (18%)	0.89
Yes, open	48 (41%)	9 (45%)	14 (48%)	25 (38%)	0.56
Coronary heart disease					0.15
No	78 (67%)	17 (85%)	17 (59%)	44 (66%)	
Yes	38 (33%)	3 (15%)	12 (41%)	23 (34%)	
Cerebrovascular accident					0.15
No	103 (89%)	20 (100%)	24 (83%)	59 (88%)	
Yes	13 (11%)	0 (0%)	5 (17%)	8 (12%)	
Diabetes mellitus					0.33
No	91 (78%)	18 (90%)	21 (72%)	52 (78%)	
Yes	25 (22%)	2 (10%)	8 (28%)	15 (22%)	
Body mass index					
Median (IQR)	25 (21–30)	23 (21–27)	25 (24–33)	26 (21–31)	0.03
Oral anticoagulants					0.29
No	100 (86%)	19 (95%)	23 (79%)	58 (87%)	
Yes	16 (14%)	1 (5%)	6 (21%)	9 (13%)	
Oral antiplatelet therapy					0.72
No	83 (72%)	15 (75%)	22 (76%)	46 (69%)	
Yes	33 (28%)	5 (5%)	7 (24%)	21 (31%)	
Nutritional risk score					0.01
< 3	58 (50%)	6 (30%)	21 (72%)	31 (46%)	
> 3	55 (47%)	14 (70%)	8 (28%)	33 (49%)	
Preoperative hemoglobin					
Median (IQR)	102 (88–117)	112 (102–132)	98 (85–117)	102 (88–111)	0.10
Intake of immunosuppressant					0.78
No	103 (89%)	18 (90%)	27 (93%)	58 (87%)	
Yes	13 (11%)	2 (10%)	2 (7%)	9 (13%)	
Intraoperative variables					
Type of operation					< 0.001
Emergency operation	90 (78%)	11 (55%)	22 (76%)	59 (88%)	
Elective operation	26 (22%)	9 (45%)	7 (24%)	8 (12%)	
Duration of surgery (min)					
Median (IQR)	155 (122–225)	260 (161–307)	172 (122–225)	149 (116–175)	< 0.001
Intraoperative blood loss (ml)					
Median (IQR)	170 (50–400)	400 (100–800)	200 (50–400)	150 (50–345)	0.03
Experience of surgeon					
Consultant general surgeon	30 (26%)	4 (20%)	8 (28%)	18 (27%)	0.80
Consultant visceral surgeon	85 (73%)	16 (80%)	20 (69%)	49 (73%)	0.69
Trainee visceral surgeon	1 (1%)	0 (0%)	1 (3%)	0 (0%)	0.42
Intraoperative use of vasopressors					0.43
No	40 (34%)	9 (45%)	10 (34%)	21 (31%)	
Yes	75 (65%)	10 (50%)	19 (66%)	46 (68%)	
Postoperative variables					
Death after colectomy					0.25
No	88 (76%)	18 (90%)	21(72%)	49 (73%)	
Yes	28 (24%)	2 (10%)	8 (28%)	18 (27%)	
Length of hospital stay (days)					
Median (IQR)	27 (17–38)	26 (15–35)	25 (18–34)	28 (19–39)	0.67

*P value is from the Pearson chi-squared test or Fisher’s exact test (when any cell frequency is < 5) for categorial variables and one-way analysis of variance (ANOVA) for continuous variables

Indicates if there is any association between demographical data in different groups

### Procedures

A total of 90 (78%) operations were performed as emergency operations. Patients in PA group had less emergency operations than in the ES and SS groups [11 patients (55%) in the PA group versus 22 patients (76%) in ES and 59 patients (88%) in SS group]. Median operative time was longer in PA group (260 min) and in the ES group (172 min) than in the SS group (149 min). Median intraoperative blood loss was higher in the PA group compared with the SS and ES group (400 ml in the PA group versus 200 ml in the ES group and 150 ml in the SS group) (Table 1). There was no significant difference in the use of vasopressors in the PA, ES, and SS groups and no difference in the experience of the surgeons performing the procedures between the three groups (Table 1).

### Outcomes

A total of 55 patients (47%) had stoma-associated complications within one year. In the univariate analysis, there was no significant difference between the PA, ES, and SS groups regarding the stoma-associated complications (Table 2). The PA and ES group had to be combined, due to the small sample size, to enable a multivariate analysis. In the multivariate analysis, no significant differences could be detected between the groups (Table 2). Due to a high 30-day mortality rate, a new analysis of the stoma-associated complications was conducted, excluding patients who had died within 30 days (Table 2). A descriptive subgroup analysis of the stoma-associated complications is presented in Table 3. No significant differences were detected in the distribution of types of complications associated with stomas.

**Table 2:** Analysis of stoma-related complications within 1 year

	Total (*N* = 116)	PA and ES group (*n* = 49)	SS group (*n* = 67)	Odds ratio (95% CI)	*P* value
Primary objective is any stoma-related complications within 1 year					
Univariate	55 (47%)	21 (43%)	34 (50%)	1.37 (0.65–2.90)	0.40
Weighted model^a^				1.48 (0.64–3.42)	0.35
Weighted model^b^				1.49 (0.62–3.56)	0.37

	Total (*N* = 94)	ES group (*n* = 29)	SS group (*n* = 67)	Odds ratio (95% CI)	*P* value
Primary objective: endileostomy versus split stoma only					
Univariate	44 (48%)	10 (34%)	34 (51%)	1.95 (0.80–4.97)	0.14

	Total (*N* = 72)	ES group (*n* = 23)	SS group (*n* = 51)	Odds ratio (95% CI)	*P* value
Primary objective: survivors only					
Univariate	36 (50%)	9 (39%)	27 (53%)	1.90 (0.70–5.31)	0.21

^a^This weighted model adjusts for all potential preoperative confounders

^b^This weighted model adjusts for all potential preoperative and intraoperative confounders

**Table 3:** Subgroup analysis of primary objective

	Total (*N* = 116)	PA group (*n* = 20)	ES group (*n* = 29)	SS group (*n* = 67)	*P* value*
Any stoma-related complications within 1 year	55 (47%)	11 (55%)	10 (34%)	34 (50%)	0.25
Stoma dehiscence	6/55 (11%)	0/11 (0%)	0/10 (0%)	6/34 (17%)	0.14
Stoma-associated wound infections	13/55 (24%)	1/11 (9%)	3/10 (10%)	9/34 (3%)	0.72
High-output stoma	15/55 (27%)	5/11 (45%)	3/10 (30%)	7/34 (20%)	0.25
High-output stoma with prerenal renal failure	6/55 (11%)	3/11 (27%)	0/10 (0%)	3/34 (9%)	0.08
High-output stoma with electrolyte imbalance	3/55 (5%)	0/11 (0%)	0/10 (0%)	3/34 (9%)	0.75
Delayed stoma movement	5/55 (9%)	1/11 (9%)	2/10 (20%)	2/34 (6%)	0.56
Other	7/55 (13%)	1/11(10%)	2/10 (20%)	4/34 (12%)	1

^*^P value is from Fisher’s exact test

Indicates if there is any association between stoma type and the outcome variable

A total of 39 patients (34%) had stoma associated complications C−D > 2, without significant differences between the groups (Table 4). Overall complication rate was high with 76% of patients experiencing complications. Patients in the PA-group had the lowest overall complication rate [11 patients (55%) in PA group, 24 patients (83%) in ES group, and 55 patients (82%) in SS group had major complications] (Table 4). In total 28 patients (24%) died in hospital. No significant differences between the groups concerning the in-hospital mortality rate could be detected (Table 4).

**Table 4:** Analysis of secondary outcomes

	Total (*N *= 116)	PA group (*n* = 20)	ES group (*n* = 29)	SS group (*n* = 67)	*P* value*
Secondary objectives					
Stoma-associated complication rate (Clavien−Dindo > II)	39/116 (34%)	5 (25%)	6 (21%)	29 (43%)	0.10
Overall Complication rate (Clavien−Dindo > II)	90/116 (76%)	11 (55%)	24 (83%)	55 (82%)	0.04
Mortality rate within 30 days	24/114 (21%)	2 (10%)	6 (21%)	16 (24%)	0.41
In-hospital mortality rate	28/116 (24%)	2 (10%)	8 (28%)	18 (27%)	0.25
Postoperative wound infection	26/116 (22%)	3 (15%)	3 (10%)	20 (30%)	0.08
Stoma closure within 1 year	49/114 (43%)	13 (65%)	7 (24%)	29 (43%)	0.02
In-hospital complication after stoma closure	9/49 (16%)	0 (0%)	1 (14%)	8 (28%)	0.06
Reoperation within 1 year without stoma closure	62/116 (53%)	6 (30%)	17 (59%)	39 (58%)	0.07
Type of reoperation					
Planned laparotomy	13 (11%)	1 (5%)	7 (24%)	5 (7%)	0.06
Unplanned laparotomy	29 (25%)	3 (15%)	5 (17%)	21 (31%)	0.17
Superficial debridement	8 (7%)	0 (0%)	2 (7%)	6 (9%)	0.52
Other	12 (10%)	2 (10%)	3 (10%)	7 (10%)	> 0.99
Any rehospitalization within 1 year	57/116 (49%)	12 (60%)	13 (45%)	32 (45%)	0.54
Stoma-associated rehospitalization	29/59 (49%)	5 (38%)	2 (15%)	22 (67%)	0.004
Increased care needs/loss of independence	53/101 (52%)	14 (71%)	14 (54%)	25 (44%)	0.30

*P value is from the Pearson chi-squared test or Fisher’s exact test (when any cell frequency is < 5)

Indicates if there is any association between stoma type and the outcome variable

Stoma closure could be achieved in 49 cases (43%). A total of 13 patients (65%) in the PA group, 7 (24%) in the ES group, and 29 (43%) in the SS group had stoma closure within 1 year. The overall complication rate after stoma closure was 16%. The highest complication rate with 28% was observed in SS group. There were no complications after closure of a loop ileostomy in the study cohort (Table 4).

Patients in PA and ES group had a significant lower stoma-associated rehospitalization rate than in SS group (38% in patients in PA-group and 15% in the ES group versus 67% of patients in the SS group needed rehospitalization) (Table 4). A total of 44 patients (38%) underwent unplanned reoperations. Of these, 5 (25%) were in the PA group, 11 (37%) were in the ES group, and 28 (42%) were in the SS group. The most common reason for unplanned reoperation were wound infections in all three groups. The most frequently conducted reoperation was an unplanned re-laparotomy. A total of 53 patients (52%) had a postoperative increased need of care. A total of 14 patients in the PA group (71%), 14 patients in the ES group (54%), and 25 patients in the SS group (44%) had a loss of independence (Table 4). An additional analysis of the stoma-related complication rate, wound infection rate, rehospitalisation rate, stoma closure rate and re-operation rate was conducted including survivors only. This additional analysis revealed a continued significantly higher stoma-associated rehospitalization rate and a higher rate of stoma closure in the SS group (Table 5).

**Table 5:** Analysis of secondary outcomes of the survivors in ES and SS groups

	Total (*N* = 72)	ES group (*n* = 23)	SS group (*n* = 51)	*P* value*
Secondary objectives				
Stoma-associated complication rate (Clavien−Dindo > II)	25/72 (35%)	5 (22%)	22 (43%)	0.08
Overall complication rate (Clavien−Dindo > II)	55 (76%)	18 (78%)	39 (76%)	> 0.99
Postoperative wound infection	19 (26%)	3 (13%)	16 (31%)	0.09
Stoma closure within 1 year	32 (44%)	7 (30%)	27 (53%)	0.01
In-hospital complication after stoma closure	7/32 (21%)	1 (14%)	6 (22%)	> 0.99
Reoperation within 1 year without stoma closure	46 (64%)	13 (57%)	33 (65%)	0.67
Type of reoperation				
Planned laparotomy	9 (13%)	6 (26%)	3 (9%)	0.02
Unplanned laparotomy	21 (29%)	3 (13%)	18 (55%)	0.09
Superficial debridement	8 (11%)	2 (9%)	6 (18%)	> 0.99
Other	8 (11%)	2 (9%)	6 (18%)	> 0.99
Any rehospitalization within 1 year	42 (58%)	13 (57%)	31 (61%)	0.92
Stoma-associated rehospitalization	21 (29%)	2 (9%)	21 (41%)	0.002
Increased care needs/loss of independence	37/72 (51%)	14 (61%)	24 (47%)	0.27

*P value is from the Pearson chi-squared or Fisher’s exact test (when any cell frequency is < 5)

Indicates if there is any association between stoma type and the outcome variable

## Discussion

Ostomy formation after right sided colectomy may be done in critically ill patients. These high-risk patients have a high in-hospital mortality rate of 24% (range 10 to 28%) and a very high morbidity of 76%. None of the strategies showed significant advantages over the others when looking at the primary outcome, stoma related complications.

Patients with primary anastomosis and formation of an upstream loop ileostomy had less complications and a higher stoma closure rate than patients in the other groups, but it was mainly performed in elective cases. Patients with end ileostomy benefited from significant lower rates of stoma associated rehospitalization. Nevertheless, the closure of an end ileostomy can only be achieved by laparotomy or laparoscopy, whereas closure of a split stoma can be performed by local mobilization. This makes it much easier to achieve closure of a split stoma. As expected, patients with split stoma therefore had higher stoma closure rates. More than 50% of the patients in this study lost their independence after stoma formation as part of right sided colectomy. In total these aspects have to be taken into account when counseling patients in case of a necessary emergency right sided colectomy and opting for a resection with discontinuity.

Nevertheless, stoma formation as part of right sided colectomy has been a rare event in the practice of these two large tertiary referral centers performing about 200 right colectomies per year. In contrast, retrospective studies suggest that in approximately 20% of patients undergoing right sided colectomy, stoma formation instead of primary anastomosis is performed [9, 23]. The stoma rate might vary based on the case mix and the proportion of emergency surgery. In general, ostomy formation is considered in case of use of high doses of vasopressors, in critically ill patients and in presence of peritonitis and perforation [9, 10].

Nevertheless, if confronted with an unstable and severely ill patient primary anastomosis with diversion will prolong operative times as seen in our data (4 h versus 2.5 h). So, the two proper rescue strategies apart from a damage control concept (which again would involve another procedure and anesthesia with a short interval) would be formation of an end ileostomy or split stoma.

Split stoma formation had a higher stoma closure rate than end ileostomy as the stoma closure is the less extensive procedure. One complication of stoma formation is the development of a high output stoma. Risk factors for the development of a high output stoma are advanced age, chronic inflammatory bowel disease (IBD), arterial hypertension, and open and emergency procedures [24].With the knowledge that stoma-related complications do not differ between the two strategies, split stoma formation offers greater advantages in presence of the mentioned risk factors, as the stoma closure is easier to perform.

More than 50% of split stomata could not be closed in our study. This is in line with the current literature, where the rate of nonclosure of temporary stomata ranges between 21% and 53% [25, 26]. Split stoma formation is associated with increased rates of stoma associated rehospitalizations. In high-risk situations and presence of specific risk factors for non-closure like anemia, impaired renal function, and metastatic disease, the additional risks associated with split stoma formation could outweigh the benefit of a rather unlikely closure [25]. If the objective is mainly to save the life of the patient and offer an easy to treat and handle ostomy, end ileostomy is the most favorable solution.

Due to the small number of patients in each group we were unable to show a significant advantage of one strategy over the other concerning the stoma-associated complication rate stoma related complications. With 65% the loop ileostomy has the highest rate of stoma closures. In the study cohort there were no in-hospital complications after closure of a loop ileostomy. However, the operations of patients who received a loop ileostomy and primary anastomosis were performed electively in 45%. The formation of a temporary loop ileostomy in case of left colonic resections is a well-established safety strategy in low rectal or otherwise high risk anastomosis [27, 28]. However, the formation of a loop ileostomy with a right sided colectomy has only been poorly described and mostly discussed as safety strategy for IBD patients with ileocolic resections [11, 29]. There is a lack of evidence whether the formation of a loop ileostomy in right-sided colectomies can reduce perioperative morbidity. On the other side a reduction of up to 55% in anastomotic leakage rate after ileostomy was reported [30]. The high stoma closure and low complication rate after closure highlights this strategy as a possible safety strategy for intermediate-risk patients who do not qualify for unprotected primary anastomosis, especially when independent risk factors for anastomotic leakage-like emergency surgery, smoking, wound classification 3 or 4, weight loss, steroid use, and prolonged operative time are present [30]. In our study cohort, the loop ileostomy was mainly performed when additional resections and second anastomosis, such as a left-sided colectomy, rectal resection or a small bowel resection, were required (see Supplementary Table 1).

Our data showed a high rate of patients that lost their independence after stoma formation. The highest rate of increased care needs was observed after formation of a loop ileostomy. Scarpa et al. reported that elderly patients have an increased need of care for stoma management compared with younger patients [31]. This needs to be factored in when talking to geriatric patients facing an emergency operation. In right-sided resections, the need of a later care home admission seems to be inevitable for a large proportion of these severely ill patients. Previous studies have shown that loop ileostomy patients seem to have a quality of life comparable to patients with end ileostomy [31].

The main limitation of the study is the small sample size. Even over a period of 14 years in two large tertiary referral centers with well-established colorectal emergency surgery practice, with a coverage area of about one million people, the number of cases was too small to draw well backed conclusions. There was a bias towards split stoma formation in most critically ill patients and end ileostomy was performed only in selected cases which is reflected by the unequal distribution of the caseload between the strategies. Further limitations are the retrospective nature and the missing of outpatient follow up data. Since patients of the coverage area are almost exclusively treated in the two institutions major complications treated elsewhere will likely not have been missed. On the other hand, mortality beyond the initial hospital admission might have been underestimated as data on outpatient deaths in care homes or the community was not available. Stoma formation in case of right sided colectomy remains a rare condition, so that it seems not feasible to conduct a prospective study. Future multicenter studies such as the ESCP collaborative colorectal audits are needed to finally be able to answer the question which technique might be superior [32].

## Conclusions

Primary anastomosis and loop ileostomy may be an option for intermediate risk patients. These patients benefit from a higher stoma closure and low complication rate after stoma closure. Patients who receive an end ileostomy benefit from lower stoma associated rehospitalization rates. In contrast, split ostomy patients showed a higher rate of stoma closure.

## Supplementary Information

Below is the link to the electronic supplementary material.

Supplementary file 1 (DOCX 13 kb)

## Data Availability

The datasets generated during and/or analyzed during the current study are not publicly available due individual privacy but specific excerpts can be obtained from the corresponding author upon reasonable request.

## Declarations

### Permission to reproduce material from other sources

No material from other sources is used.
