# Supplementary material for: Stoma associated complications after diverting loop ileostomy, end ileostomy or split stoma formation after right sided colectomy—a retrospective cohort study (StoComSplit Analysis)
Source: Tech Coloproctol. 2024 Jun 12;28(1):68. doi: 10.1007/s10151-024-02945-z (PMC11169016; doi:10.1007/s10151-024-02945-z)
Supplement: Supplementary file 1 — (DOCX 13 kb) [file 10151_2024_2945_MOESM1_ESM.docx]

**Supplementary Table 1: Primary Causes for the Creation of Diverting Loop Ostomies**

|  | **N (%)** |
| --- | --- |
| **Additional colon resection** | **12/20 (60%)** |
| **Additional small bowel resection** | **3/20 (15%)** |
| **As a component of anastomosis revision** | **2/20 (10%)** |
| **Other** | **3/20 (15%)** |
